# Opportunities for prevention and intervention with young children: lessons from the Canadian incidence study of reported child abuse and neglect

**DOI:** 10.1186/1753-2000-7-4

**Published:** 2013-02-13

**Authors:** Barbara Fallon, Jennifer Ma, Kate Allan, Melanie Pillhofer, Nico Trocmé, Andreas Jud

**Affiliations:** 1 Factor-Inwentash Faculty of Social Work, University of Toronto, 246 Bloor Street W, Toronto, Ontario M5S 1V4, Canada; 2 Clinic for Child and Adolescent Psychiatry/Psychotherapy, University Hospital Ulm, Ulm, Germany; 3 Centre of Research for Children and Families, McGill University, Montreal, Québec, Canada

**Keywords:** Child welfare, Child maltreatment, Infants, Young parents, Referral source, Decision-making, Ongoing services

## Abstract

**Background:**

The most effective way to provide support to caregivers with infants in order to promote good health, social, emotional and developmental outcomes is the subject of numerous debates in the literature. In Canada, each province adopts a different approach which range from universal to targeted programs. Nonetheless, each year a group of vulnerable infants is identified to the child welfare system with concerns about their well-being and safety. This study examines maltreatment-related investigations in Canada involving children under the age of one year to identify which factors determine service provision at the conclusion of the investigation.

**Methods:**

A secondary analysis of the Canadian Incidence Study of Reported Child Abuse and Neglect *CIS-2008* (PHAC, 2010) dataset was conducted. Multivariate analyses were conducted to understand the profile of investigations involving infants (n=1,203) and which predictors were significant in the decision to transfer a case to ongoing services at the conclusion of the investigation. Logistic Regression and Classification and Regression Trees (CART) were conducted to examine the relationship between the outcome and predictors.

**Results:**

The results suggest that there are three main sources that refer infants to the Canadian child welfare system: hospital, police, and non-professionals. Infant maltreatment-related investigations involve young caregivers who struggle with poverty, single-parenthood, drug/solvent and alcohol abuse, mental health issues, lack of social supports, and intimate partner violence. Across the three referral sources, primary caregiver risk factors are the strongest predictor of the decision to transfer a case to ongoing services.

**Conclusions:**

Multivariate analyses indicate that the presence of infant concerns does not predict ongoing service provision, except when the infant is identified with positive toxicology at birth. The opportunity for early intervention and the need to tailor interventions for specific caregiver risk factors is discussed.

## Introduction and Background

The most effective way to provide support to caregivers with infants in order to promote good health, social, emotional and developmental outcomes is the subject of numerous debates in the empirical literature. Each province/territory in Canada adopts a different approach which range from universal to targeted programs. Nonetheless, each year a group of vulnerable infants is identified to the Canadian child welfare system with concerns about their well-being and safety.

In Canada, both non-professionals and professionals who have concerns about child maltreatment can make a referral to a child welfare agency. The child welfare agency determines whether or not an initial investigation will occur after they receive the referral. If there is an initial investigation, child welfare workers typically determine whether or not maltreatment has occurred, and whether or not the family will receive voluntary or non-voluntary child welfare services. Workers may decide to provide ongoing child welfare services at the conclusion of the investigation, meaning that the child and/or family will have an open case file with the child welfare agency, and will maintain ongoing contact with an agency employee until it is determined that supportive services are no longer necessary.

The primary objectives of this paper are (1) to examine the decision to provide ongoing child welfare services to infants identified to the child welfare system using a Canadian national dataset, (2) to understand the clinical factors that influence the decision to provide ongoing child welfare services to infants and their caregivers, and (3) to situate the findings in a public health context and understand opportunities for prevention and intervention in families struggling with maltreatment-related issues.

Infants are the most vulnerable subset of children involved with the child welfare system given their dependency on a caregiver to take care of their daily needs, and their inability to protect themselves from any form of harm [1,2]. In 2008, children under the age of one were the most likely to be the subject of maltreatment-related investigations in Canada with rates of investigations decreasing with age [3]. This pattern was also observed in 1998 and 2003 [3]. Given the high incidence of investigations involving infants, understanding the factors that impact child welfare service delivery to infants and their families is important.

The rate of infant maltreatment related investigations in Canada in 2008 was 51.81 per 1,000 children, a non-significant increase from the 2003 rate of investigation [3]. A dramatic increase in the rate of infant investigation occurred earlier, between 1998 and 2003 when the rate increased from 17.23 to 49.54 [3]. This increase was consistent with an overall increase in the rate of all child maltreatment investigations in Canada [3]. Various factors may have contributed to this increase in investigations including changes in detection, reporting and investigation practices [3]. Furthermore, legislative changes introduced provincially expanded reporting criteria to include cases where a child had not yet been harmed, but where a risk of future maltreatment was evident [4].

Differential service response models have been recently introduced in several Canadian jurisdictions, which permit workers to conduct family needs assessments as opposed to full investigations in cases where the risk level is found to be low to moderate, including British Columbia [5], Alberta [6], and Ontario [7]. Cases involving infants, however, are generally considered high-risk due to the vulnerability of this population [2]. A study found that caregivers of infants were more likely to have a drug, alcohol, learning or medical problem and to be experiencing domestic violence compared to caregivers of older children involved with the child welfare system [8]. Federally-mandated developmental screening in the United States suggests that children who become involved with the child welfare system in infancy present developmental delays more often than children in the general population [9]). In the 2003 Canadian Incidence Study of Reported Child Abuse and Neglect, workers noted few developmental concerns and positive toxicology at birth or substance abuse birth defects in 93% of investigations involving infants [10]. However, several studies suggest that children involved with the child welfare system may be under-identified for developmental difficulties [9,11,12].

Currently, at the point at which infants come into contact with the child welfare system, there is at minimum risk factors present that could potentially impact the child’s social, emotional, cognitive, intellectual or physical development [3]. In Canada, infants are most often brought to the attention of the child welfare system by health professionals and second most often by police, often while law enforcement is responding to an incident of domestic violence [2]. Preventive programs, which may begin prenatally, may help to support parents and mitigate risk factors for maltreatment prior to the birth of the child (e.g., Nurse Family Partnership Program) [13,14].

It is important to understand the clinical profile of families with risk factors for maltreatment, as this may assist in preventing harm to children, supporting well-being, and preventing intrusive child welfare intervention. Early prevention of maltreatment is a public health issue, and programs that are tailored and responsive to the needs of at-risk families are necessary. Preventing maltreatment will in turn help to prevent the consequences of maltreatment, such as childhood injury and developmental difficulties, and it will also lessen the case volume at child protection agencies. Overall, investing in early identification and prevention is beneficial for individuals and families as well as society as a whole, with efforts in the early years producing excellent economic returns and other positive outcomes [15].

## Methods

A secondary analysis of the Canadian Incidence Study of Reported Child Abuse and Neglect *CIS-2008*[16] dataset was conducted. Ethics approval for this study was provided by University of Toronto, McGill University and University of Calgary. Please refer to Chapter 2 in the CIS-2008 Major Findings Report for more detailed information about methods [3]. The CIS-2008 dataset contains information about key clinical factors collected during routine child maltreatment investigations [3]. A multi-stage sampling design was employed to first obtain a representative sample of 112 child welfare agencies selected from 412 child welfare service areas in Canada, and then to sample cases within these agencies [3]. Maltreatment-related cases opened for investigation at the agencies between October 1st and December 31st^a^ were eligible for inclusion [3]. Three months was considered to be the optimal period for participation and compliance with study procedures. The final sample selection stage involved identifying children who had been investigated due to concerns related to possible maltreatment. Maltreatment-related investigations included situations where there were concerns that a child may have already been abused or neglected as well as situations where there was no specific concern about past maltreatment but where the risk of future maltreatment was being assessed. A maltreatment investigation occurred when there was an allegation made about a known or suspected past incident of abuse or neglect. Risk investigations were conducted when there were no allegations or suspicions of past abuse or neglect, but rather the concern was the risk of future maltreatment. Together, maltreatment and risk investigations are referred to as “maltreatment-related investigations” throughout this paper.

In most jurisdictions cases were counted as families, so procedures were developed to determine which specific children in each family had been investigated for maltreatment-related concerns. In jurisdictions outside of Québec, children were eligible for inclusion in the final study sample if the worker investigated a maltreatment-related concern (i.e., investigation of possible past incident(s) of maltreatment or assessment of risk of future maltreatment). In Québec, children were eligible for inclusion in the final study sample if the case was “retained”^b^ with maltreatment-related classification codes.

### Data collection instruments

Workers in the sampled child welfare agencies completed the three-page data collection instrument at the conclusion of their initial maltreatment-related investigation. The CIS-2008 data collection instrument was based on the instrument used in previous cycles of the CIS. In preparation for the CIS-2008, the instrument was revised and validated through a case file validation study, validation focus groups, and a reliability study (please see Trocmé et al., 2010 for details). The data collection instrument included clinical information that workers would have collected as part of their initial investigation. Workers were trained on completing the instrument, and were asked to use their clinical judgment to respond to the questions. Data collected included: referral source; type of investigation (maltreatment or risk only); type of abuse and neglect investigated; level of substantiation; functioning concerns for the children and risk factors for their caregivers; income source; housing information; and information about short-term service dispositions. Key clinical variables were included in the analysis in order to reflect an ecological model and to determine the relative contribution of clinical variables to the decision to provide ongoing services (please see Table 1). Completion rates were over 98% on most items; this high item completion rate can be attributed to the design of the instrument and the verification procedures [3].

**Table 1:** Variable definitions

**Outcome Variable**	**Measurement**	**Description**
Transferred to Ongoing Service	Dichotomous variable	Workers were asked to indicate whether the investigation would be opened for ongoing child welfare services at the conclusion of the investigation.
	Transfer to ongoing service(1)	
	Close case (0)	
Predictor Variables		
Primary Caregiver Age	Categorical variable	Workers were asked to indicate the age category of the primary caregiver.
	18 years and under (1)	
	19 to 21 years (2)	
	22 to 30 years (3)	
	31 to 40 years (4)	
	41 years and up (5)	
Primary Caregiver Risk Factors	Nine dichotomous variables	Workers could note up to nine risk factors for the primary caregiver. Risk factors were: alcohol abuse, drug/solvent abuse, cognitive impairment, mental health issues, physical health issues, few social supports, victim of domestic violence, perpetrator of domestic violence, and history of foster care/group home.
	Suspected or confirmed concern (1)	
	No or unknown (0)	
Child Functioning	Six dichotomous variables	Workers could note up to eighteen functioning concerns for the investigated child, indicating whether the concern had been confirmed, suspected, was not present or it was unknown to the worker. This analysis examined six age-appropriate concerns, including: attachment issues, intellectual/developmental disability, failure to meet developmental milestones, Fetal Alcohol Syndrone/Fetal Alcohol Effects (FAS/FAE), positive toxicology at birth, and physical disability.
	Suspected or confirmed concern (1)	
	No or unknown (0)	
No Second Caregiver in the Home	Dichotomous variable	Workers were asked to describe up to two caregivers in the home. If there was only one caregiver described there was no second caregiver in the home.
	No Second caregiver in the home (1)	
	Second caregiver in the home (0)	
Primary Income	Categorical variable	Workers were asked to indicate the primary source of the primary caregiver’s income.
	Full time employment (1)	
	Part time/seasonal employment (2)	
	Other benefits/ unemployment (3)	
	No income (4)	
Household Hazards		Workers were asked to note if the following hazards were present in the home at the time of the investigation: accessible weapons, accessible drugs, production/trafficking of drugs, chemicals/solvents used in drug production, other home injury hazards, and other home health hazards.
	At least one household hazard (1)	
	No household hazards (0)	
Household Regularly Runs Out of Money	Dichotomous variable	Workers were asked to note if the household regularly runs out of money.
	Noted (1)	
	Not Noted (0)	
Number of Moves	Categorical variable	Number of moves reflects the number of moves the household had experienced in the past six months.
	No moves (0)	
	One move (1)	
	Two or more moves (2)	
Type of Investigation	Maltreatment investigation (1)	Workers were asked to indicate whether the investigation was for an incident of maltreatment or if it was a risk investigation only.
	Risk-only investigation (2)	
Referral Source		
Source of Allegation/ Referral	Nine dichotomous variables	Workers were asked to indicate all sources of referral that were relevant for each investigation. This includes separate and independent contact with the child welfare agency. Workers could note up to nineteen referral sources for the investigation. Referral source variables were collapsed into nine categories: non-professional referral sources (custodial parent, non-custodial parent, relative, neighbour/friend), community or social services (social assistance worker, crisis service/shelter, community/recreation centre, community health nurse, community physician, community mental health professional, community agency), hospital, school, other child welfare service, day care centre, police, anonymous, and other.
	Noted (1)	
	Not Noted (0)	

### Study sample

The *CIS-2008* sampling procedures yielded a final sample of 15,980 children investigated because of maltreatment-related concerns (i.e., incident of maltreatment or risk assessment). This analysis focused on investigations involving children under the age of one year (n=1,203), examining whether the case was transferred to ongoing services at the conclusion of the investigation. The sample was further divided into three categories of referral sources: hospital referrals; police referrals; and non-professional referrals. The categories were selected for practical reasons, because the majority of infant investigations were referred by one of these referral sources. Almost one quarter of investigations involving infants were referred by hospital personnel (23%). Approximately 22% of infant investigations were referred by the police. Non-professional referral sources comprised 23% of investigations involving infants. This implies that approximately 68% of all infant investigations were referred to by hospital personnel, police, or non-professionals. The remaining infant investigations were referred by other professional sources (e.g., community or social services, day care centre, etc.; please see Table 1 for complete list). Workers could list multiple referral sources, if there were multiple independent contacts with the child welfare agency.

Two sets of weights were applied to the data to derive national annual estimates. First, results were annualized to estimate the volume of cases investigated by each study site over the entire year. To account for the non-proportional sampling design, regional weights were then applied to reflect the size of each site relative to the child population in the region from which the site was sampled. Annualization weights are based on service statistics from the study sites; these service statistics include an unknown number of “duplicate” cases, or in other words, children or families reported and opened for investigation two or more times during the year. Although each investigation represents a new maltreatment-related concern, confusion arises if these investigations are interpreted to represent an “unduplicated” count of children. To avoid this confusion, the CIS-2008 uses the term “child investigations” rather than “investigated children” [3]. The final weighted sample for child maltreatment investigations involving infants was 17,339.

## Measures

### Outcome variable: transferred to ongoing services

Workers were asked to indicate whether the case would be opened for ongoing child welfare services at the conclusion of the investigation. The decision to transfer a case to ongoing services is a dichotomous variable.

### Predictor variables

Key clinical variables representing an ecological model of child maltreatment were examined to determine the relative contribution of clinical variables. Clinical variables were chosen based on empirical literature of factors related to child maltreatment or risk of child maltreatment. These included child functioning concerns, caregiver risk factors, and household characteristics. The operational definitions and codes used in the analysis are provided in Table 1.

## Analysis plan

All analyses were conducted using SPSS, version 20.0. Descriptive analyses were conducted to explore the characteristics of investigations involving children under the age of one year (infants). Annualization and regionalization weights were applied in the descriptive analysis to derive national annual incidence estimates. National incidence estimates were calculated by dividing the weighted estimates by the child population. Bivariate analyses were also conducted to examine the relationship between the outcome variable and each relevant predictor variable. The sample weight was applied in the bivariate analyses to adjust for inflation of the chi-square statistic by the size of the estimate by weighting the estimate back down to the original sample size.

Multivariate analyses were conducted to understand the profile of investigations involving infants (n=1,203) and which predictors were significant in the decision to transfer a case to ongoing services at the conclusion of the investigation. Logistic Regression and Classification and Regression Trees (CART) were conducted to examine the relationship between the outcome and predictors. Unweighted data were used in all models. The weights were not applied in the multivariate analyses to ensure unbiased results. Logistic Regression was completed to predict the outcome variable of transfers to ongoing services. Logistic regression is appropriate for the type of data that is found in social and behavioural research, where many of the dependent variables of interest are dichotomous and the relationships among the independent and dependent variables are not necessarily linear [17]. Logistic regression uses maximum likelihood estimation after the dependent variable has been transformed into a logit variable. The logit variable is the log of the odds of the dependent variable occurring. Through this means, logistic regression can estimate the probability of an event occurring [17].

Only significant predictor variables at the bivariate level (p<.05) were included in the logistic regression model. The choice of cutoff point for the decision to provide ongoing services was set at .45 which reflects the proportion of investigations transferred to ongoing services for this population. The cutoff point represents the classification rate and ensures accuracy in the analysis. Predictors with a significant relationship (p < .05) to the decision to transfer the case to ongoing child welfare services were retained from the first model. The model was then run with this smaller set of significant predictors (p<.05).

For the Classification and Regression Trees (CART) analysis, the objective was to understand which predictors (caregiver, child, household, and case characteristics) determine the decision to transfer a case to ongoing services among specific referral sources. Through recursive partitioning, the CART methodology develops hierarchical binary classification trees [18]. All variables were included the CART analysis given the possibility that a predictor variable may be significantly related to the outcome variable for a subset of the sample regardless of that predictor’s relationship with the outcome variable for the whole sample [18].

To attain a more comprehensive understanding of the predictors of transfers to ongoing services among investigations involving infants, three models were developed. The sample was divided into three categories of referral sources: hospital referrals; police referrals; and non-professional referrals (reports from custodial and non-custodial parents, relatives, and/or neighbours/friends). The categories were selected for practical reasons based on the results of the univariate analysis. As such, the first model examined infant investigations referred to the child welfare system from hospitals, the second model examined investigations referred by the police, and the third model examined investigations referred by a non-professional referral. All of the models included caregiver characteristics (age, caregiver risk), child characteristics (child functioning), household characteristics (no second caregiver, income, household hazards, household regularly runs out of money, and number of moves), and case characteristics (type of investigation). All models were developed to determine how caregiver, child, household, and case characteristics interact to predict transfers to ongoing services among the three referral sources to examine

The minimum size for parent node (n=50) and child node (n=20) were specified prior to analyses in order to decrease the likelihood of overfitting the data. Nodes refer to the subsamples resulting from partitioning the sample. The parent node refers to the minimum size of the subsample to split and the child node refers to the minimum size for the resulting node after the split. Furthermore, cross-validation was completed to assess the generalizability and stability of the final tree models [18]. A ten-fold cross-validation procedure was conducted, in which the sample was randomly divided into ten subsamples and ten models were produced which alternately excluded one of the subsamples. The cross-validation process determines an average risk estimate across models. A comparison risk estimate of the final model against the average risk estimate indicates how close the final model is to other potential models and determines whether the final model is a good representation of the available data [18].

## Results

The results revealed important descriptive information about the profile of child welfare investigations in Canada in 2008, involving infants and their families. Almost a quarter of investigations involving infants were referred by hospital personnel (3,935 investigations, 22.7%). Similarly, 22.1% of these investigations were referred by the police (3,833 investigations). Non-professional referral sources comprised 23% of investigations involving infants (3,986 investigations). Approximately 15% of the infant investigations were referred by other community/health or social services (2,601 investigations). A minority of investigations were referred by schools (739, 4.3%), other child welfare services (1,194, 6.9%), and day care centres (81, 0.5%). The referral sources of maltreatment-related investigations involving infants are presented in Table 2.

**Table 2:** Referral sources of maltreatment-related investigations involving infants in Canada in 2008 (n = 17,339)

	**Frequency**	**%**
Non-Professional	3,986	23.0%
Other Community/Health or Social Services	2,601	15.0%
Hospital (any personnel)	3,935	22.7%
School	739	4.3%
Other Child Welfare Service	1,194	6.9%
Day Care Centre	81	0.5%
Police	3,833	22.1%
Anonymous	920	5.3%
Other	473	2.7%

*Workers could endorse multiple referral sources, if there were multiple independent contacts with the child welfare agency. Totals do not add up to 100%.

Most primary caregivers were under the age of 30 years. Approximately 13% (2,173) of the caregivers were 18 years old and under and 19.8% (3,408) were between the ages of 19 and 21 years old. Almost half (7,679, 44.6%) of the caregivers were 22 to 30 years old. About 20% (3,488) of the caregivers were 31 to 40 years old. A minority (482, 2.8%) of the caregivers were 41 years old or older. At least one caregiver risk factor was noted in 76.6% (13,283) of infant investigations. The most common caregiver risk factor identified was victim of domestic violence, with 39.1% (6,778) primary caregivers identified as a victim of domestic violence. The next most common caregiver risk factor identified was few social supports (6,142, 35.4%) followed by mental health issues (4,703, 27.1%). Drug/solvent abuse (4,356, 25.1%) and alcohol abuse (3,511, 20.3%) were noted risks for some of the caregivers. Investigating workers also identified history of foster care/group home (2,781, 16.0%), cognitive impairment (2,095, 12.1%), and physical health issues (1,299, 7.5%) as risk factors.

Of the relevant child functioning concerns noted for infants the most common concern was positive toxicology at birth (1,253 investigations, 7.2%). Fetal Alcohol Syndrome/Fetal Alcohol Effects (FAS/FAE) was identified in 480 investigations (2.8%). Investigating workers identified failure to meet developmental milestones in 626 investigations (3.6%), and attachment issues in 443 investigations (2.6%). Physical disability was identified as a concern in 369 investigations (2.1%). Intellectual or developmental disability was a child functioning concern in 401 investigations (2.3%).

Approximately one third of investigations involved single-parent households (5,535 investigations, 31.9%). Over half of the primary caregivers involved in infant investigations relied on other benefits or unemployment as their primary source of income (10,553 investigations or 60.9%). About 26.5% (4,587 investigations) had no income. Some of the primary caregivers were employed full-time (1,378 investigations, 7.9%) while a minority had part-time or seasonal employment (821 investigations, 4.7%). In a small proportion of investigations, the worker identified at least one hazard present in the household (1,683 investigations, 9.7%) or identified that the household regularly ran out of money (2,515 investigations, 14.5%). Most investigations involved families that had not moved in the past six months (5,440 investigations or 31.4%) or moved once in the past six months (4,658 investigations or 26.9%).

Of all the investigations involving infants, 10,656 represented a maltreatment investigation (61.5%) and 6,684 represented a risk investigation (38.5%). Of all maltreatment-related investigations in this sample, neglect was identified as the overriding concern in almost one third of cases (4,913 investigations, 28.1%). Exposure to intimate partner violence was identified as the primary concern in 3,917 investigations (22.6%). In a small proportion of maltreatment-related investigations in this sample, the overriding concern was physical abuse (1,190 investigations, 6.8%), emotional maltreatment (537 investigations, 3.1%), or sexual abuse (99 investigations, 0.6%). In 7,044 investigations (40.6%), the case was transferred to ongoing services. The clinical characteristics of infant maltreatment-related investigations are reported in Table 3.

**Table 3:** Clinical concerns of maltreatment-related investigations involving infants in Canada in 2008 (n = 17,339)

	**Frequency**	**%**
Primary Caregiver Age		
18 Years and Under	2,173	12.6%
19 to 21 Years	3,408	19.8%
22 to 30 Years	7,679	44.6%
31 to 40 Years	3,488	20.2%
41 Years and Older	482	2.8%
Primary Caregiver Risk Factors		
Alcohol Abuse	3,511	20.3%
Drug/Solvent Abuse	4,356	25.1%
Cognitive Impairment	2,095	12.1%
Mental Health Issues	4,703	27.1%
Physical Health Issues	1,299	7.5%
Few Social Supports	6,142	35.4%
Victim of Domestic Violence	6,778	39.1%
Perpetrator of Domestic Violence	1,437	8.3%
History of Foster Care/Group Home	2,781	16.0%
At Least One Functioning Concern	13,283	76.6%
Child Functioning Concerns		
Attachment Issues	443	2.6%
Intellectual/Developmental Disability	401	2.3%
Failure to Meet Developmental Milestones	626	3.6%
FAS/FAE	480	2.8%
Positive Toxicology at Birth	1,253	7.2%
Physical Disability	369	2.1%
No Second Caregiver in the Home	5,535	31.9%
Primary Income		
Full-time	1,378	7.9%
Part-time/Seasonal	821	4.7%
Other Benefits/Unemployment	10,553	60.9%
No Income	4,587	26.5%
At Least One Household Hazard	1,683	9.7%
Household Regularly Runs Out of Money	2,515	14.5%
Number of Moves		
No Moves	5,440	31.4%
One Move	4,658	26.9%
Two or More Moves	2,843	16.4%
Type of Maltreatment		
Physical Abuse	1,190	6.8%%
Sexual Abuse	99	0.6%%
Neglect	4,913	28.1%
Emotional Maltreatment	537	3.1%
Exposure to Intimate Partner Violence (IPV)	3,917	22.6%
Risk	6,684	38.5%

A multiple logistic regression model was fit to predict the probability of being opened for ongoing services at the conclusion of maltreatment-related investigations involving infants using clinical concerns as predictors. The results of the model are summarized in Table 4. Overall, the regression model was able to predict 67.8% of the cases as they were classified correctly. The omnibus tests of model coefficients (X^2^(24) = 137.96, *p* < .001) shows that the model is significant.

**Table 4:** Probability of being opened for ongoing services at the conclusion of maltreatment-related investigations involving infants in Canada in 2008 (n = 17,339)

**Predictors**	**b**	***SE***	***wald***	***e***^**b**^
Primary Caregiver Age (18 Years and Under)				
19 to 21 Years	-.33	.29	1.28	.72
22 to 30 Years	-.58	.26	4.88	.56*
31 to 40 Years	-.78	.30	6.82	.46**
41 Years and Older	.31	.55	.33	1.37
At Least One Caregiver Risk Factor	1.19	.24	24.70	3.28***
Child Functioning Concerns				
Attachment Issues	.65	.60	1.20	1.32
Intellectual/Developmental Disability	.53	.90	.34	1.69
Failure to Meet Developmental Milestones	.10	.62	.03	1.10
FAS/FAE	.61	.70	.77	1.84
Positive Toxicology at Birth	1.16	.36	10.31	3.19**
Physical Disability	-.26	.76	.12	.77
No Second Caregiver in the Home	-.31	.19	2.74	.74
Primary Income (Full-time)				
Part-time/Seasonal	.01	.52	.00	1.01
Other Benefits/Unemployment	.12	.34	.12	1.13
No Income	-.03	.37	.01	.97
At Least One Household Hazard	1.08	.30	13.46	3.03***
Household Regularly Runs Out of Money	.39	.22	2.17	1.47
Number of Moves (No Moves)				
One Move	.07	.19	.14	1.07
Two or More Moves	.68	.24	8.36	1.97**
Type of Maltreatment (Physical Abuse)				
Sexual Abuse	-.70	1.61	.19	.50
Neglect	-.77	.36	4.44	.46*
Emotional Maltreatment	-.03	.51	.00	.97
Exposure to Intimate Partner Violence	-.50	.36	1.96	.61
Risk	-.32	.35	.85	.73
	−2 Log Likelihood			Omnibus Tests of Model Coefficients (X^2^)
Model	880.52			137.96***

* p < .05, ** p < .01, *** p < .001.

The results indicate that most of the primary caregiver characteristics (age, caregiver risk factors) are significant predictors of being opened for ongoing services at the conclusion of a maltreatment-related investigation involving infants. The presence of at least one risk factor among primary caregivers increases the likelihood of being transferred to ongoing services by a factor of 3.28 (Exp(B) = 3.28, *p* < .001). In comparison to being a primary caregiver aged 18 years and under, being a caregiver between the ages of 22 to 30 years or 31 to 40 years decreases the likelihood of being transferred to ongoing services by a factor of 0.56 (Exp(B) = 0.56, *p* < .05) or 0.46 (Exp(B) = 0.46, *p* < .01) respectively. The only significant child functioning concern predicting transfers to ongoing services is positive toxicology at birth. When this concern was noted by investigating workers, it increases the likelihood of service provision by a factor of 3.19 (Exp(B) = 3.19, *p* < .01). Of the household characteristics included in the analysis, the presence of at least one household hazard significantly increases the likelihood of transfers to ongoing services by a factor of 2.95 (Exp(B) = 2.95, *p* < .001). In comparison to investigations involving families who did not move during the past year, investigations involving families who moved two or more times are significantly more likely to be opened for ongoing services at the conclusion of the investigation by a factor of 1.97 (Exp(B) = 1.97, *p* < .01). Lastly, maltreatment type was included in the analysis to control for the influence of the type of maltreatment being investigated. Investigations of neglect are less likely to be transferred to ongoing services in comparison to investigations of physical abuse by a factor of 0.46 (Exp(B) = 0.46, *p* < .05).

CART analysis was conducted to determine how child welfare workers decided which families received ongoing services at the conclusion of investigations using all characteristics which included: caregiver characteristics (age and caregiver functioning), child characteristics (child functioning), household characteristics (no second caregiver, primary income, household hazards, household regularly runs out of money, and number of moves), and case characteristics (type of investigation). Three models were developed to examine the predictors of transfers to ongoing services among hospital referrals, police referrals, and non-professional referrals. Cross-validation was conducted using all characteristics to assess the generalizability and stability of the final CART model.

**Figure 1:**
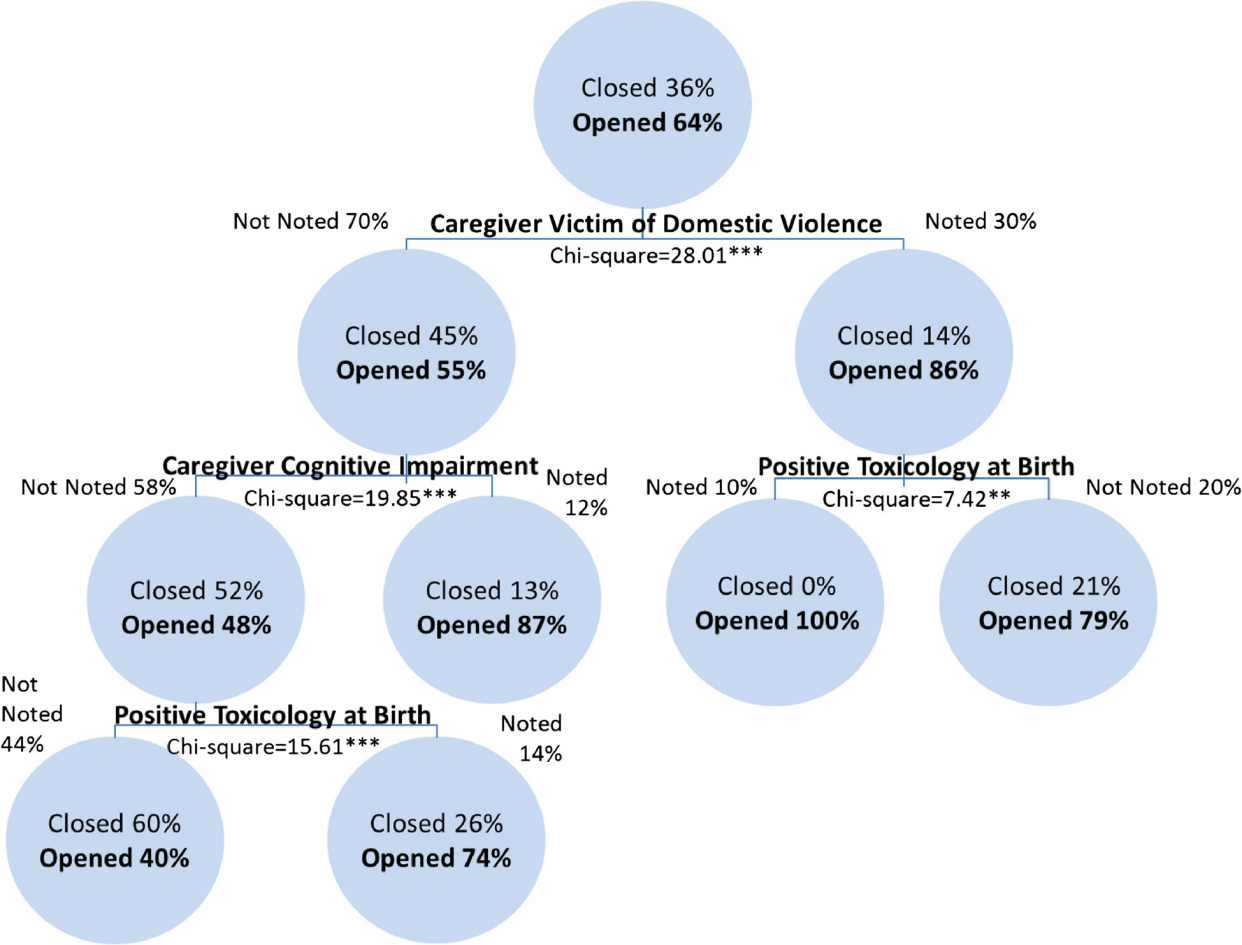
Transfers to ongoing services among hospital referred investigations involving infants in Canada in 2008 (classification rate = 73.1%).

Of the infant investigations referred by hospital personnel, the identification of the primary caregiver as a victim of domestic violence is the most significant predictor of the provision of ongoing services. The next best predictor of service provision among investigations where domestic violence was noted is infant positive toxicology at birth. Of the investigations where the caregiver was not a victim of domestic violence, the next best predictor of case transfer is primary caregiver cognitive impairment. Among investigations where the caregiver was not identified as a victim of domestic violence and was not identified with a cognitive impairment, positive toxicology at birth predicts case transfer to ongoing services. While the risk estimate of the cross-validation analysis of .329 indicates that the category predicted by the model is wrong for 32.9% of the cases, the classification table indicates that the model classifies 73.1% of the investigations correctly. Figure 1 shows the results of the CART analysis of hospital referred infant investigations.

**Figure 2:**
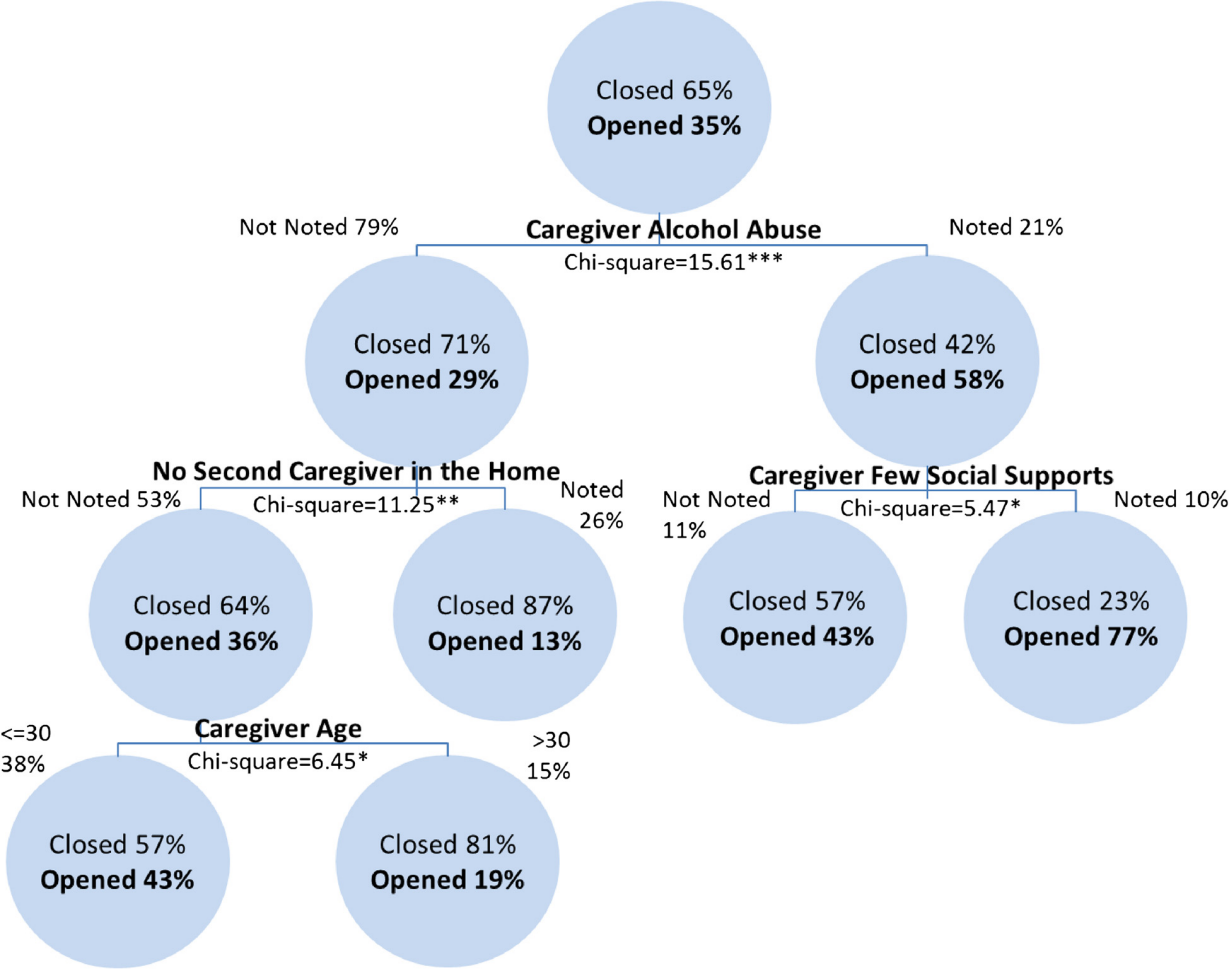
Transfers to ongoing services among police referred investigations involving infants in Canada in 2008 (classification rate = 70.3%).

Among investigations involving infants referred by police, primary caregiver alcohol abuse is the most significant predictor of transfers to ongoing services at the conclusion of maltreatment-related investigations. The next best predictor of service provision where caregiver alcohol abuse is a concern is caregiver few social supports. Of investigations where alcohol abuse and lack of social supports are not noted risk factors, the next best predictor of transfers to ongoing services is single-parenthood. Where investigations do not note alcohol abuse or single-parenthood, the next best predictor of transfers to ongoing services is caregiver age. Investigations involving caregivers younger than 30 years of age are more likely to be transferred for ongoing services. The risk estimate of the cross-validation analysis of .366 indicates that the category predicted by the model is incorrect for 36.6% of the cases. However, the classification table indicates that the model correctly classifies 70.3% of the investigations. The results of the CART analysis of police referred infant investigations are presented in Figure 2.

**Figure 3:**
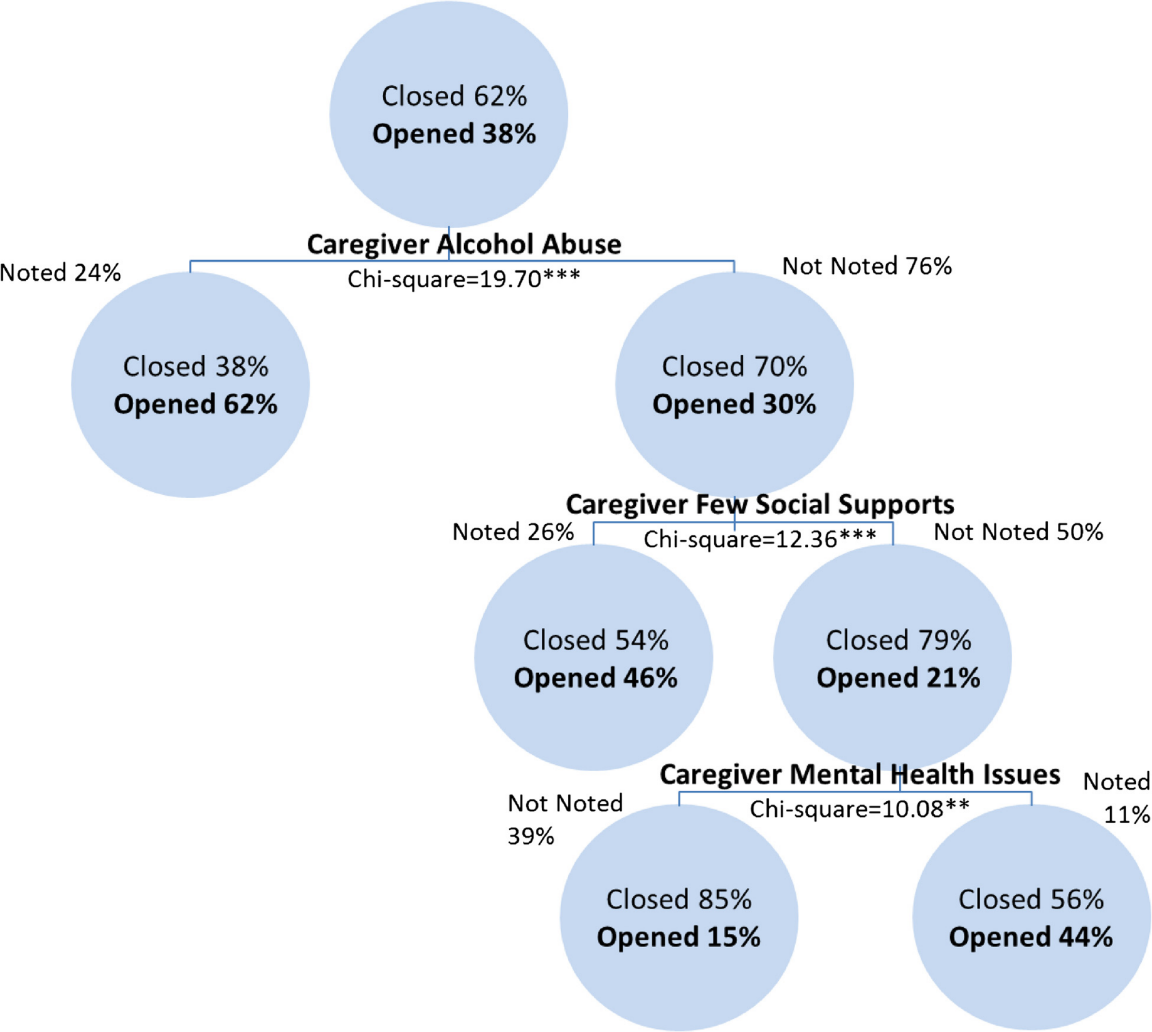
Transfers to ongoing services among non-professional referred investigations involving infants in Canada in 2008 (classification rate = 68.3%).

For infant investigations referred by non-professional referral sources, primary caregiver alcohol abuse is the most significant predictor of transfers to ongoing services at the conclusion of an investigation. Of the investigations where alcohol abuse is not a concern, the next best predictor of transfers to ongoing services is caregiver few social supports. Where alcohol abuse and lack of social supports are not noted concerns, the next best predictor of transfers to ongoing services is caregiver mental health issues. The risk estimate of the cross-validation analysis of .358 demonstrates that the category predicted by the model is incorrect for 35.8% of the cases while the results of the classification analysis show that the model classifies 68.3% of the investigations correctly. Figure 3 presents the results of the CART analysis for non-professional referral sources.

## Discussion

This study used a Canadian national child welfare dataset to examine the profile of infants and their families who are the subject of maltreatment-related investigations in order to identify which factors impact the decision to provide ongoing services at the conclusion of the investigation. Several findings have significance to the child welfare field. When examining all investigations involving infants, significant predictors of case transfer include caregiver age (young caregivers more likely to be transferred), caregiver risk factors, infant positive toxicology at birth, and two or more moves in the past 12 months. These findings provide a broad profile of clinical characteristics driving service provision.

The CIS collects data on up to 19 potential sources of referral. For investigations involving infants in Canada, there were three main sources of referral: hospitals, police, and non-professionals. The results of the analysis of the decision to provide ongoing services by referral source indicate that caregiver risk factors are most predictive of service provision. The only child functioning concern found to be a significant predictor of transfers to ongoing services was positive toxicology at birth when controlling for other clinical concerns of the investigation. In addition, single-parent households were also a significant predictor of the decision to provide ongoing services.

While the risk factors noted for caregivers drive the decision to provide ongoing services at the conclusion of an investigation, there are different clinical profiles for infant maltreatment-related investigations observed depending on the source of referral. Hospital referrals have a high rate of opening (65%) and whether or not the caregiver is a victim of domestic violence is the strongest predictor of service provision. Other factors predicting transfers to ongoing services among hospital referrals are caregiver cognitive impairment and positive toxicology at birth. For police referrals which involve investigations of intimate partner violence the most significant predictor of service provision is caregiver alcohol abuse followed by single-parent households, the level of social support that the primary caregiver has in the community, and the age of the caregiver as younger caregivers are more likely to be transferred to ongoing services. Finally, the strongest predictor of service provision for infant investigations referred by non-professional referral sources is caregiver alcohol abuse, followed by few social supports and a caregiver mental health issue. The results of the current analysis reflect the findings of previous studies which indicate that concerns relating to the caregiver such as substance use, lack of social support, mental health issues, young age and domestic violence are risk factors for infant maltreatment [19-21].

### Implications

Maltreatment prevention programs have the potential to identify family-level risk factors at an earlier stage and to circumvent the need for child welfare involvement with young families. The success of these programs depends on the degree to which program design and implementation is tailored to the specific needs of families [22]. This study demonstrates that child welfare workers are noting a variety of clinical needs for the caregivers of infants, including substance abuse issues, mental health issues, lack of social supports and domestic violence concerns. The lack of universal early prevention and intervention programs in Canada has resulted in missed opportunities for preventing maltreatment in families with risk factors. Understanding the needs of families involved with the child welfare system may contribute to the development of prevention programs which are tailored to respond to the different needs of at-risk families in Canada.

The early identification of at-risk families may help to improve the dynamic between the child protection system and Canadian families, as programs assessing the needs of families replace protection investigations. As the developmental impact of maltreatment in early childhood becomes clearer, the need for prevention programs to mitigate maltreatment risk factors becomes more urgent [19,23].

### Limitations

Data from the CIS-2008 are collected directly from the investigating worker and are not independently verified. These data only represent the concerns that present during an average six week investigation period. Additional concerns for the child and the caregiver could arise after the initial investigation. Weighted estimates do not account for seasonal variation in maltreatment typologies.

## Conclusion

This study described the decision to provide ongoing child welfare services at the conclusion of investigations involving infants, focusing on the different clinical profiles that emerge depending on the referral source for the investigation. It found that caregivers of infants are struggling with a number of issues including poverty, single-parenthood, drug/solvent and alcohol abuse, mental health issues, lack of social supports, and violence. The functioning of the caregiver is the strongest determinant of child welfare involvement across referrals from hospitals, police, and non-professional referral sources. Preventative strategies and early interventions in key areas could improve the outcomes for infants and their families. The opportunity to target interventions for the different clinical profiles of the families that emerge for this very vulnerable population is evident.

## Endnote

^a^In several Aboriginal jurisdictions, this adjustment was made to accommodate late enrolment of some Aboriginal sites.

^b^Agencies in Quebec use a structured phone screening process whereby approximately half of all referrals are “retained” for evaluation. In Québec, the CIS sampled retained maltreatment-related reports that involved cases that were not already open.

## Competing interests

The authors declare that they have no competing interests.

## Authors’ contributions

BF conducted the study and provided conceptual direction. JM conducted the analyses and drafted the manuscript. KA conducted the literature review. MP reviewed the draft and provided substantive feedback. NT provided conceptual direction. AJ conceived of the study and provided feedback. All authors read and approved the final manuscript.
